# Inflammation and platelet hyperresponsiveness in coronary artery disease and the influence of Talin-1/αIIbβ3-mediated bidirectional signaling pathway

**DOI:** 10.3389/fphar.2025.1535182

**Published:** 2025-03-20

**Authors:** Shengnan Shi, Jiaming Gao, Yehao Zhang, Min Zhan, Zhanfei Tan, Peili Wang, Jianhua Fu, Jianxun Liu

**Affiliations:** ^1^ Beijing Key Laboratory of Pharmacology of Chinese Materia Medica, Institute of Basic Medical Sciences of Xiyuan Hospital, China Academy of Chinese Medical Sciences, Beijing, China; ^2^ Department of Encephalopathy, Xiyuan Hospital, China Academy of Chinese Medical Sciences, Beijing, China; ^3^ Wangjing Hospital, China Academy of Chinese Medical Sciences, Beijing, China; ^4^ National Clinical Research Center for Chinese Medicine Cardiology, Xiyuan Hospital, China Academy of Chinese Medical Sciences, Beijing, China

**Keywords:** coronary heart disease, platelet hyperreactivity, Talin-1 and αIIbβ3-mediated bidirectional signaling pathway, platelet transcriptome, interleukin

## Abstract

**Background:**

While platelet hyperreactivity constitutes an independent risk factor for major adverse cardiovascular events (MACEs) in coronary artery disease, its molecular underpinnings remain poorly characterized. Recent advances in transcriptomic profiling have revealed potential associations with specific RNA signatures. Through systematic bioinformatics analysis of differential gene expression patterns and pathway activation in CHD patients, this study aims to elucidate key molecular regulators of platelet hyperactivity, establishing a theoretical framework for developing precision therapeutic strategies to mitigate post-CHD complications.

**Methods:**

This randomized controlled study included 16 CHD patients and 16 healthy controls. Inflammation markers, platelet aggregation function, and CD62p levels were assessed using flow cytometry. Mitochondrial morphology and organelles were observed using scanning electron microscopy and transmission electron microscopy. Genes related to symptom alteration between CHD patients and healthy controls were identified using the criteria of p < 0.05. The molecular correlations of these genes were analyzed using a comprehensive perspective that included Gene Ontology (GO) biological process and Kyoto Encyclopedia of Genes and Genomes (KEGG) pathway analyses. Western blot and correlation analyses were also conducted to validate the expression and diagnostic value of the DEGs.

**Results:**

CHD patients exhibited alterations in platelet organelles ultrastructure, heightened platelet activation and aggregation, and disturbance of the inflammatory equilibrium. RNA sequencing demonstrated distinct changes in the gene expression profiles of circulating platelets from CHD patients. The increase in platelet activation and aggregation could be partially associated with the upregulation of the Talin-1 and αIIbβ3 proteins expression.

**Conclusion:**

Abnormal transcription and platelet activation occur after CHD onset, and upregulation of the Talin-1/αIIbβ3-mediated bidirectional signaling pathway are the primary pathological features.

**Clinical Trial Registration:**

https://www.chictr.org.cn/, identifier ChiCTR2100041998.

## Introduction

Coronary heart disease (CHD) remains the leading cause of mortality and morbidity worldwide (Martin et al., 2024). The clinical trajectory of CHD critically depends on the stability of atherosclerotic plaques, with vulnerable plaques—characterized by thin fibrous caps, necrotic lipid cores, and inflammatory cell infiltration—serving as the primary substrate for acute thrombotic events (Otsuka et al., 2014). At the site of coronary plaque rupture, platelets adhere to collagen exposed to the bloodstream, accumulate, and facilitate thrombosis that restricts coronary perfusion, ultimately resulting in myocardial ischemia (Baaten et al., 2024). Current antiplatelet therapies mitigate thrombosis by selectively targeting specific platelet receptors (such as P2Y12 or GPIIb/IIIa), modulating molecular signaling pathways (such as adenosine diphosphate-mediated signaling), or through the irreversible inhibition of the cyclooxygenase (COX) enzyme mediated by aspirin (Gawaz et al., 2023). Nevertheless, recurrent ischemic events continue to pose a significant clinical challenge, particularly in patients with residual high-risk plaque features detected by intravascular imaging (Clayton et al., 2005; Xie et al., 2014). Inadequate platelet inhibition or platelet hyperreactivity may elevate the risk of thrombosis through enhanced cross-talk with unstable plaque microenvironments (Stone et al., 2013).

Personalized antiplatelet therapy based on platelet function measurement has received a great deal of attention (such as multiple-electrode aggregometry or thromboelastography) (Sibbing et al., 2019). Platelet transcriptome analysis, as an innovative technology, is being utilized to uncover novel dimensions of platelet biology (Middleton et al., 2019; Plé et al., 2012). A substantial number of transcripts and mRNAs are present in platelets, where they regulate platelet transcription, translation, and biological processes. The associated biological functions and molecular regulatory mechanisms play an important role in the prognosis of CHD (Manne et al., 2020; Gnatenko et al., 2003; Suresh et al., 2014). The purpose of the present study was to identify the key targets and signaling pathways of platelet in patients with CHD by screening the differential gene expression of platelets, so as to provide a reference for drug development.

## Methods

### Study design

**FIGURE 1:**
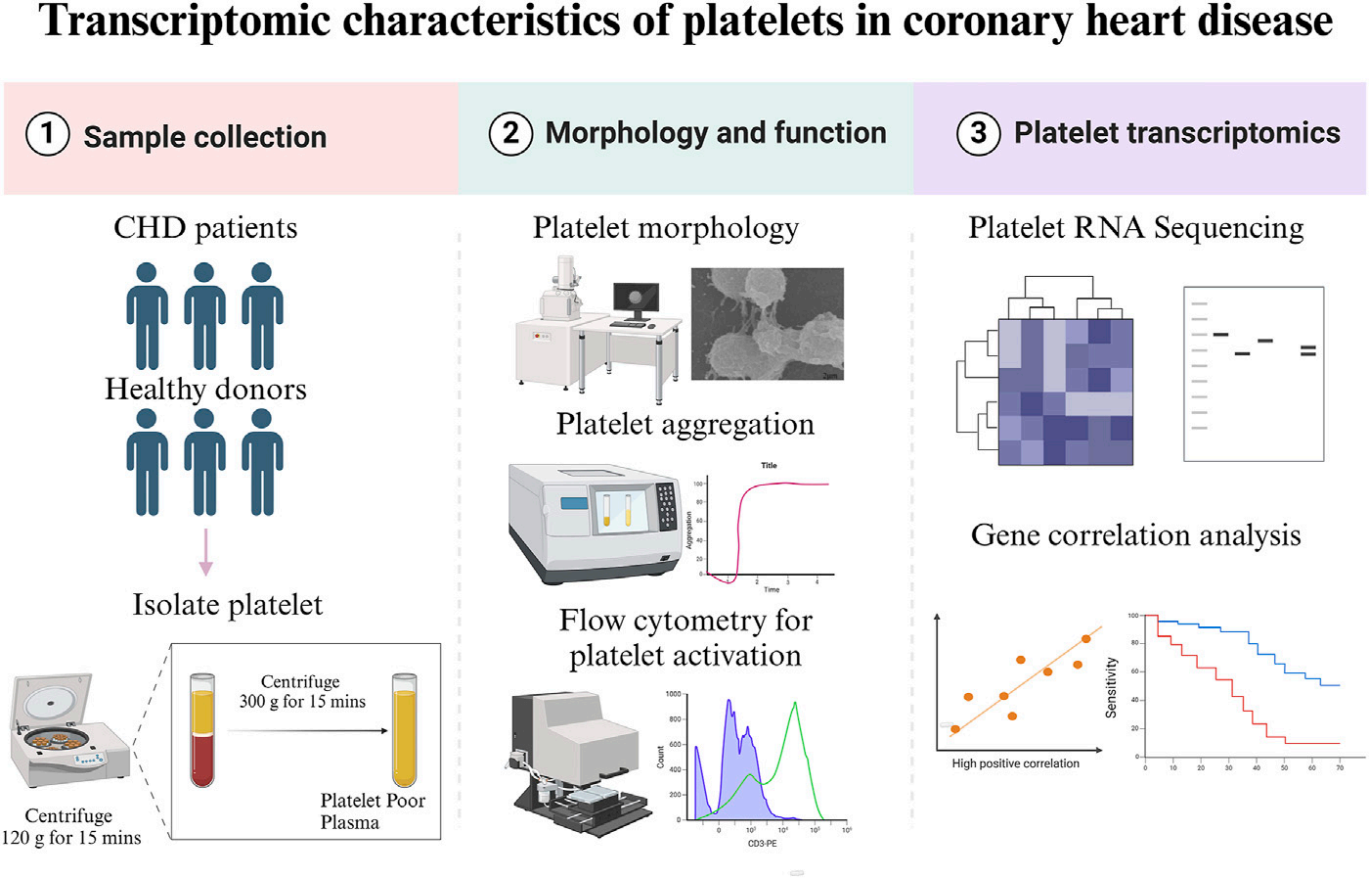
Summary of methodologies.

The study was approved by the Ethics Committee of Xiyuan Hospital (IRB#: 2020XLA057) and was performed in accordance with the Declaration of Helsinki. All participants signed informed consent before recruitment. Patients with CHD were recruited from Xiyuan Hospital, China Academy of Chinese Medical Sciences, Beijing between December 2020 and December 2021. Inclusion criteria encompassed an age range of 40–85 years and the absence of the use of antiplatelet and anticoagulant drugs within the week prior to enrollment. Exclusion criteria were recent acute coronary syndrome, malignant arrhythmia, myocarditis, hypertrophic cardiomyopathy, severe valvulopathy, pulmonary embolism, malignant tumor, autoimmune disorders, severe infectious diseases, trauma, recent surgical procedures, severe hepatic and renal dysfunction, or malignant hematopathy. Healthy age- and sex-matched donors (controls) were enrolled from the Physical Examination Department of Xiyuan Hospital. The baseline demographic, medical history, medication history, biochemical parameters, risk factor, lifestyle and other information of the patients were collected. For a summary of the methodologies, please refer to Figure 1.

### Incident CHD Ascertainment

In accordance with 2019 ESC Guideline for the diagnosis and management of chronic coronary syndromes, the patients with CHD included in this study were as follows: 1. Coronary angiography was carried out to determine the existence of coronary stenosis and acute, transient myocardial ischemia-hypoxia syndrome caused by increased myocardial load; 2. Patients who were hospitalized for ACS or coronary revascularization and discharged in a stable condition (Knuuti et al., 2019).

### Platelet isolation

Human platelets were isolated from the venous blood of CHD patients and healthy donors. Citrated whole blood (1:9 v/v) was centrifuged at 120 *g* for 15 min to obtain platelet-rich plasma. Platelet-rich plasma was centrifuged at 300 *g* for 15 min after removal of the supernatant.

### Platelet aggregation

Aggregation was measured using an AG800 aggregometer (Techlink, Beijing, China) at 37°C with stirring. Washed platelets were stimulated with different agonists (including ADP 5 μmol/L, arachidonic acid 0.25 mg/mL, and collagen 5 μg/mL) to evaluate the degree of aggregation.

### Flow cytometry for platelet activation

Washed platelets were preincubated with mouse anti-human PAC1-FITC specific for activated integrin αIIbβ3 and anti-P-selectin-PE specific for P-selectin (both from BD Biosciences) for human platelets. After 10 min at 37°C, the samples were analyzed via flow cytometry.

### Flow cytometry to measure inflammatory factor release

The inflammatory factors IL-1β, IL-2, IL-4, IL-5, IL-6, IL-8, IL-10, IL-12p70, IL-17, IFN-α, IFN-γ, and TNF-α were detected with corresponding detection kits (R701001, R701002, R701003, R701004, R701005, R701006, R701007, R701008, Ralsecare, Shandong, China) according to the manufacturer’s instructions and analyzed with an LSRFortessa instrument (BD). The samples were mixed with buffers, antibody capture beads and detection antibodies (25 μL each) and incubated in the dark at room temperature for 2 h. Then, 25 μL of PE fluorescent secondary antibody was added, and the sample was incubated in the dark at room temperature with shaking for 0.5 h. After this, 1 mL of wash buffer was added, and the sample was centrifuged at 500 *g* for 5 min; finally, the microspheres were resuspended in 300 µL wash buffer and tested on a flow cytometer.

### Western blotting

Platelets were lysed in RIPA buffer containing 0.1% sodium dodecyl sulfate, 1% Triton X-100, 0.5% sodium deoxycholate, 150 mM NaCl, 50 mM Tris-Cl (pH 8.0), and 1 mM EDTA supplemented with protease inhibitors (Roche). Proteins were separated via electrophoresis using 6%, 7.5% and 10% (w/v) polyacrylamide gels containing 0.1% (w/v) sodium dodecyl sulfate (SDS) and transferred to polyvinylidene difluoride membranes. The membranes were incubated with antibodies against Talin (Abcam, ab92537), Integrin α2 (Abcam, ab119992), Integrin β3 (Abcam, ab133557), RIAM(Abcam, ab157808), p-PI3K(Proteintech, 66009-1-lg), PI3K(ZEN BIO, 341468), p-AKT (CST, 4060), AKT (CST, 9272), RAP1(CST, 2399) and β-actin (Abcam, ab8226) at 1:1000 and detected via chemiluminescence with an ECL Western blotting detection system.

### Scanning and transmission electron microscopy

Platelets were adhered to coverslips coated with polylysine, fixed in 2.5% glutaraldehyde for 48 h at room temperature and analyzed with a 120 kV JEOL electron microscope at 80 kV.

### RNA preparation and sequencing

Total RNA was isolated from platelets using TRIzol reagent according to the manufacturer’s instructions. The concentration, integrity and purity of the total isolated RNA were assessed using an Agilent 2100 Bioanalyzer (Agilent Technologies, Santa Clara, CA, USA). The libraries were established using a TruSeq Stranded mRNA LT Sample Prep Kit (Illumina, San Diego, CA, USA). RNA-seq and analysis were performed by OE Biotech Co. The clean reads were mapped to the human genome (GRCh38) using HISAT2 default parameters. The FPKM value of each gene was calculated using Cufflinks, and the read counts of each gene were obtained via HTSeqcount. Differential expression analysis was performed using the DESeq R package. A P value <0.05 and a fold change >2 or <0.5 were set as the thresholds for significantly differential expression. Volcano plots and heatmaps were generated to show the expression patterns of different genes in different samples. GO and KEGG pathway enrichment analyses of the DEGs were performed using R based on the hypergeometric distribution.

### Statistical analyses

Statistical analyses were performed using Prism 7 (GraphPad, San Diego, CA). A t-test (mean and standard deviation) was used to determine the statistical significance of differences between two groups. Correlation analysis was performed using Minitab (State College, PA, USA). P < 0.05 was considered to indicate statistical significance.

## Results

### Clinical characteristics of patients in the CHD and healthy donor cohort

The clinical characteristics and laboratory data of the 32 participants in the study are shown in Table 1. CHD patient cohort were well matched to healthy donors in terms of age, sex, body mass index, and blood pressure. The CHD patients included 8 men and 8 women with a mean age of 59.88 years and a mean body mass index (BMI) of 24.55 kg/m^2^. Approximately one-third of patients had experienced hypertension and hyperlipidemia, and close to a quarter had a previous MI. Compared with healthy donors, CHD patients had higher levels of UA and Fbg but lower levels of APTT and TT. The results of echocardiography showed that the left atrial diameter, left internal diameter and right anteroposterior diameter were increased in CHD patients, while the left ventricular ejection fraction was decreased.

**TABLE 1:** Patient characteristics.

Characteristics	CHD patients (n = 16)	Healthy donors (n = 16)	p value for trend
Age, yrs	59.88 ± 10.77	53.44 ± 6.89	0.055
Female, n (%)	8 (50%)	9 (56.25%)	1.00
Body mass index, kg/m2	24.55 ± 1.80	24.37 ± 3.14	0.846
Blood pressure, mmHg			
Systolic	122.88 ± 8.43	126.38 ± 8.37	0.248
Diastolic	70.00 ± 6.78	81.94 ± 7.51	0.000
Comorbidity, n (%)			
Prior MI	4 (25%)	0	
Prior PCI	3 (18.75%)	0	
Prior CABG	1 (6.25%)	0	
Prior stroke	1 (6.25%)	0	
Hypertension	6 (37.5%)	0	
Diabetes mellitus	2 (12.5%)	0	
Hyperlipoidemia	5 (31.25%)	0	
Current smoker	2 (12.5%)	3 (18.75%)	
Drinking history	7 (43.75%)	5 (31.25%)	
Previous antiplatelet medication, n (%)			
Aspirin	7 (43.75%)	0	
Clopidogrel	2 (12.5%)	0	
Current Medication,n (%)			
Beta-blocker	2 (12.5%)	0	
Nitrates	2 (12.5%)	0	
Statins	12 (75%)	0	
Calcium-channel blocker	5 (31.25%)	0	
ACEI	1 (6.25%)	0	
ARB	1 (6.25%)	0	
Hypoglycemic drugs	2 (12.5%)	0	
Trimetazidine	1 (6.25%)	0	
Laboratory data			
WBC, 10^9^/L	6.33 ± 1.34	8.35 ± 11.90	0.506
RBC, 10^12^/L	4.61 ± 0.40	4.67 ± 0.40	0.724
HGB, g/L	141.50 ± 13.13	142.13 ± 11.54	0.887
PLT, 10^9^/L	259.25 ± 51.14	226.44 ± 42.53	0.058
PCT, %	0.26 ± 0.05	0.24 ± 0.04	0.173
PDW, fL	10.96 ± 1.11	12.26 ± 2.62	0.084
MPV, fL	10.03 ± 0.52	10.50 ± 1.09	0.136
P-LCR, %	24.70 ± 4.43	28.52 ± 8.46	0.120
TG, mmol/L	1.38 ± 0.93	1.07 ± 0.32	0.22
TC, mmol/L	4.45 ± 0.97	4.48 ± 0.77	0.921
HDL-C, mmol/L	1.23 ± 0.29	1.23 ± 0.25	0.979
LDL-C, mmol/L	2.45 ± 0.70	2.73 ± 0.50	0.208
BUN, mmol/L	14.95 ± 7.26	13.52 ± 2.59	0.465
CR, mmol/L	73.38 ± 20.81	67.93 ± 10.90	0.361
UA,mmol/L	357.38 ± 116.38	252.64 ± 53.86	0.003
PT, sec	10.84 ± 0.49	10.91 ± 0.41	0.669
PTA, %	110.81 ± 6.58	113.97 ± 17.65	0.508
INR	0.94 ± 0.04	0.94 ± 0.04	0.786
APTT, sec	26.77 ± 1.76	28.18 ± 1.61	0.025
TT, sec	17.05 ± 0.61	17.41 ± 0.33	0.047
Fbg, g/L	3.13 ± 0.72	2.61 ± 0.39	0.020
Echocardiography			
LAD, mm	36.44 ± 3.97	33.06 ± 2.57	0.008
IVST, mm	8.56 ± 0.63	8.25 ± 0.86	0.249
LVD, mm	49.00 ± 4.62	46.06 ± 3.11	0.043
LVPWT, mm	8.50 ± 0.52	8.25 ± 0.86	0.325
RVPWT, mm	4	4	
LVEF, %	62.13 ± 4.56	67.06 ± 3.92	0.003

### CHD patients exhibit increased platelet surface P-selectin and PAC-1 expression and platelet aggregation

**FIGURE 2:**
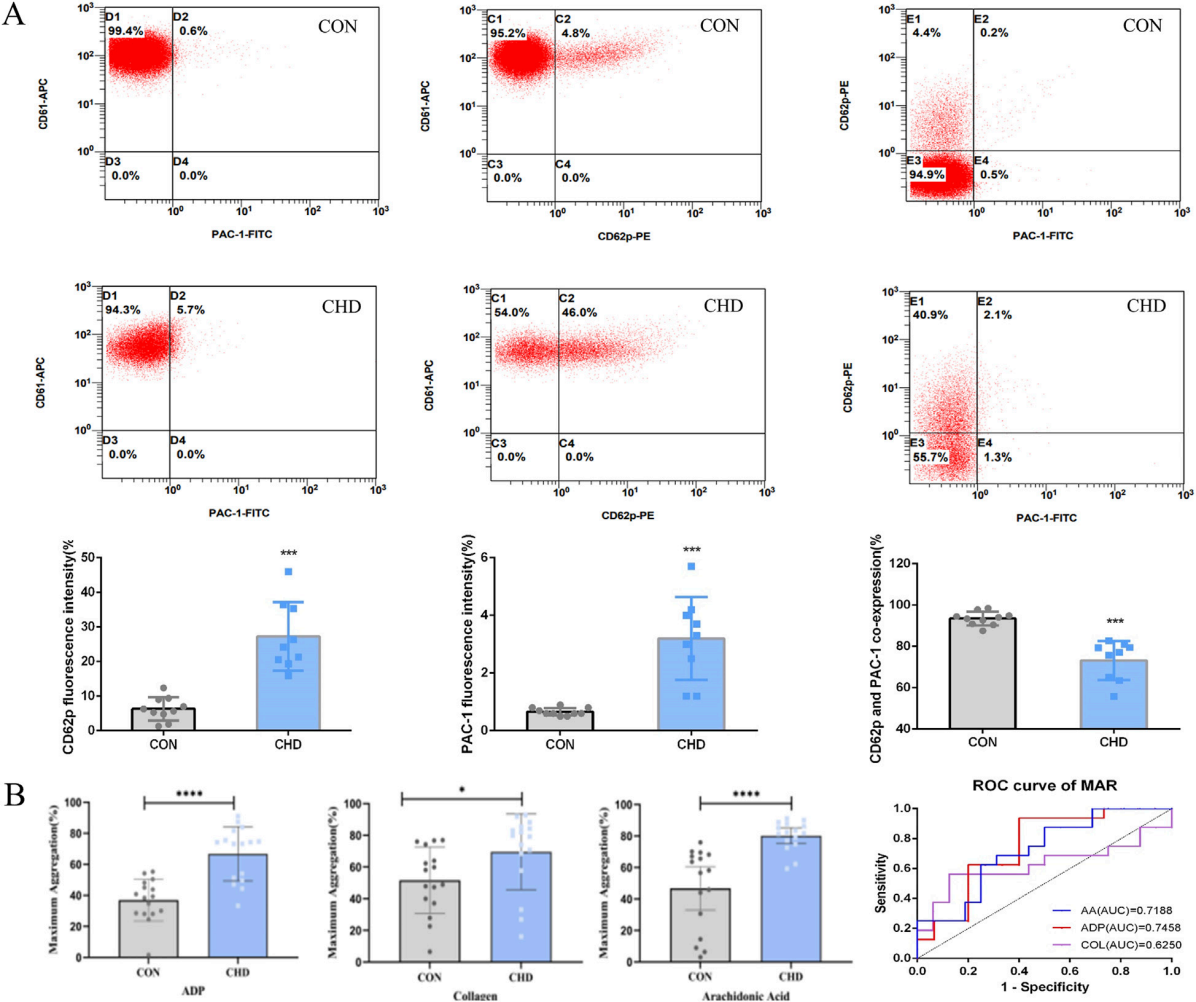
Platelets from CHD patients are hyperreactive. **(A)**, Platelet CD62p and PAC-1 activation (n = 9); **(B)**, Platelet aggregation (n = 16). Data are shown as the mean fluorescence intensities ± SDs of the control *p < 0.05, **p < 0.01, ***p < 0.001.

Next, to confirm activation of platelets in CHD cohort, we measured the expression of CD62p and PAC-1 as the gold indicators with flow cytometry. The results showed that in CHD patients, the expression of the platelet-activating glycoproteins CD62p and PAC-1 were significantly increased (Figure 2A). Interestingly, in CHD patients, the ratio of platelets with CD62p and PAC-1 coexpression decreased. However, in CHD patients, platelet activation may be induced mainly through the combination of CD62p receptors.

Since we observed evidence of increased platelet activation in all CHD patients (Figure 2A), we next assessed platelet aggregation. Platelet aggregation in response to platelet agonists (ADP, collagen, and arachidonic acid) was significantly greater in CHD patients than in healthy donors (Figure 2B).

### Platelet morphology in CHD patients showed a higher activation state accompanied by obvious morphological abnormalities of organelles

**FIGURE 3:**
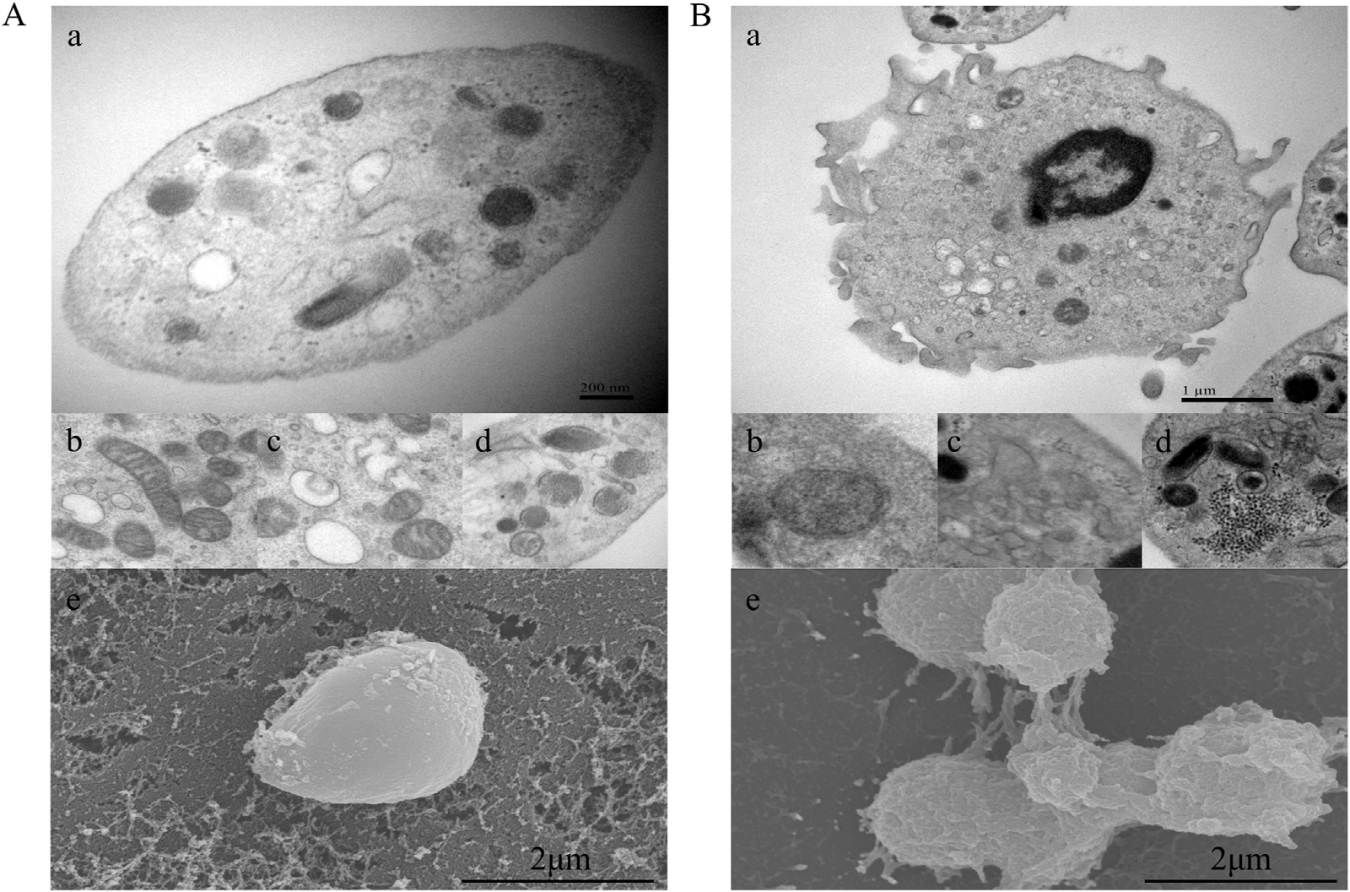
Platelet morphological characteristics in CHD patients. **(A)**, Platelets from healthy donors; **(B)**, platelets from CHD patients. a, microtubular coils around the periphery of the platelet; b, mitochondria; c, open canicular system; d, alpha and dense granules; e, platelet outer membrane status. Magnification, ×20000.

Morphological observation of purified platelets was performed to determine concordance between phenotypic changes and subsequent transcriptomic changes. We examined platelet morphology and ultrastructure during CHD using transmission electron microscopy (TEM) and scanning electron microscopy (SEM). Three samples of CHD patients with significantly elevated CD62p were randomly selected from the above flow cytometry results for electron microscopy, for comparison, three matched healthy donors were observed. Representative images are shown in Figures 3A, B. The open canicular system (OCS), alpha and dense granules, mitochondria, and microtubular coils around the periphery were observed in platelets from both healthy donors and CHD patients. Compared with those in the normal group, the platelets in the CHD group exhibited obvious pseudopod formation, and the mitochondrial bilayer membrane structure in the normal group was clear and numerous; moreover, the number of mitochondria in the platelets in the CHD group decreased sharply, and part of the membrane structure was dissolved. The OCS in the normal group was clear and vacuole-like, while the OCS in the disease group was filled with secretions. In addition, the platelets in the CHD group were more likely to form secretory granules. SEM showed that the outer membrane of the platelets was flat in the normal group and more convex and clustered in the disease group. The formation of platelet pseudopodia indicates the increase of aggregation and adhesion capacity, the increase of particle release indicates the activation of receptor pathways related to its activation, and the mitochondria are the organelles that supply the energy required for platelet activation, and its morphological changes suggest that the energy metabolism of platelets in CHD patients is abnormal.

### The balance of the inflammatory system is disrupted in CHD patients

**FIGURE 4:**
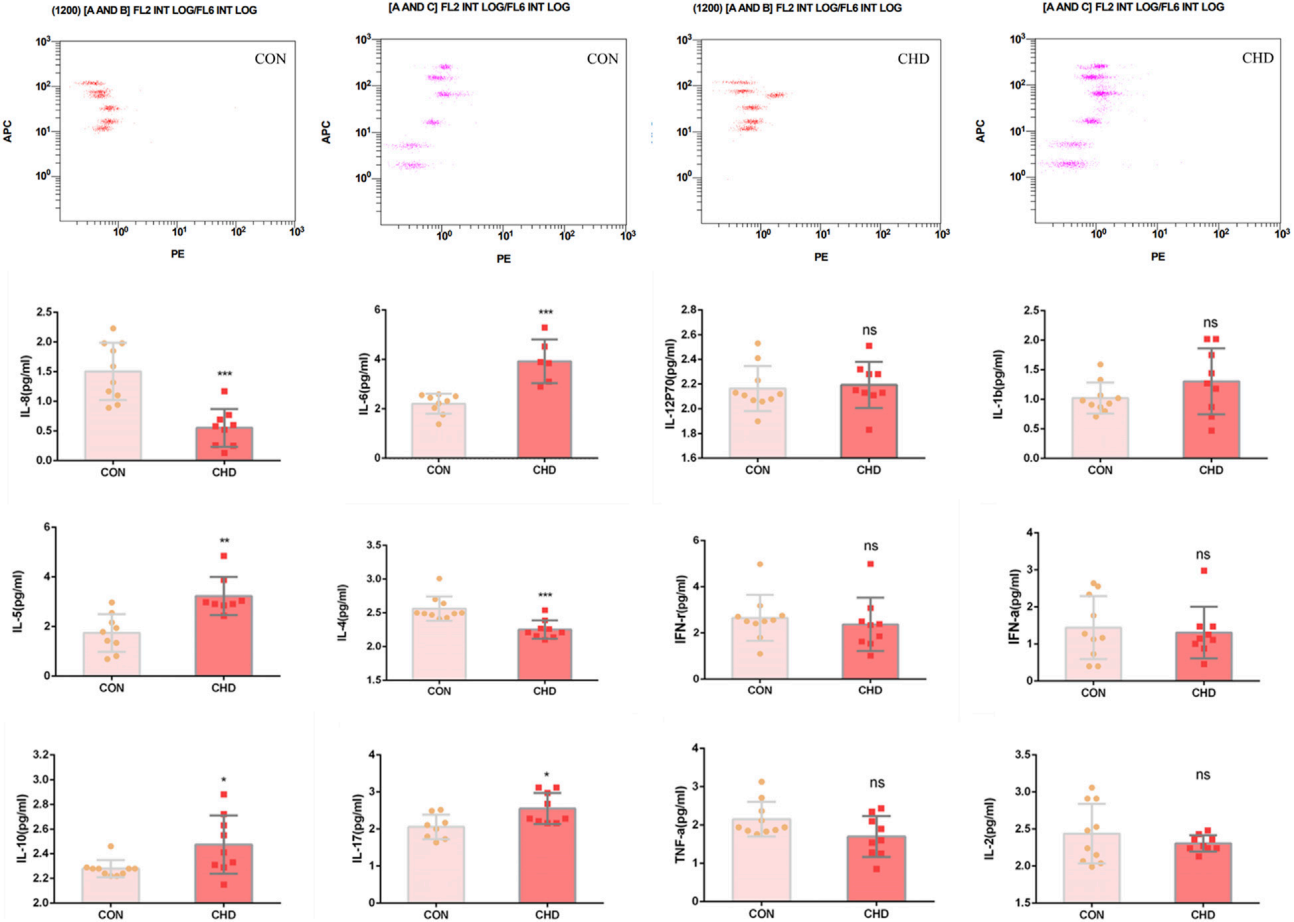
Plasma levels of the inflammatory factors IL-8, IL-6, IL-12, IL-1b, IL-5, IL-4, IFN-γ, IFN-α, IL-6, IL-17, TNF-α and IL-2. The data shown are the means ± SDs. *p < 0.05, **p < 0.01, ***p < 0.001.

The levels of IL-8 and IL-4 in the plasma of CHD patients decreased, and the levels of IL-6, IL-5, IL-10 and IL-17 increased, indicating that there is still disordered inflammation regulation balance in CHD patients and that these inflammatory factors also stimulate platelets and abnormal physiological activities. Moreover, there was no significant difference in the concentrations of the other inflammatory factors (Figure 4).

### Activation of the Talin-1 and αIIbβ3-mediated bidirectional signaling pathway is upregulated in CHD patients

**FIGURE 5:**
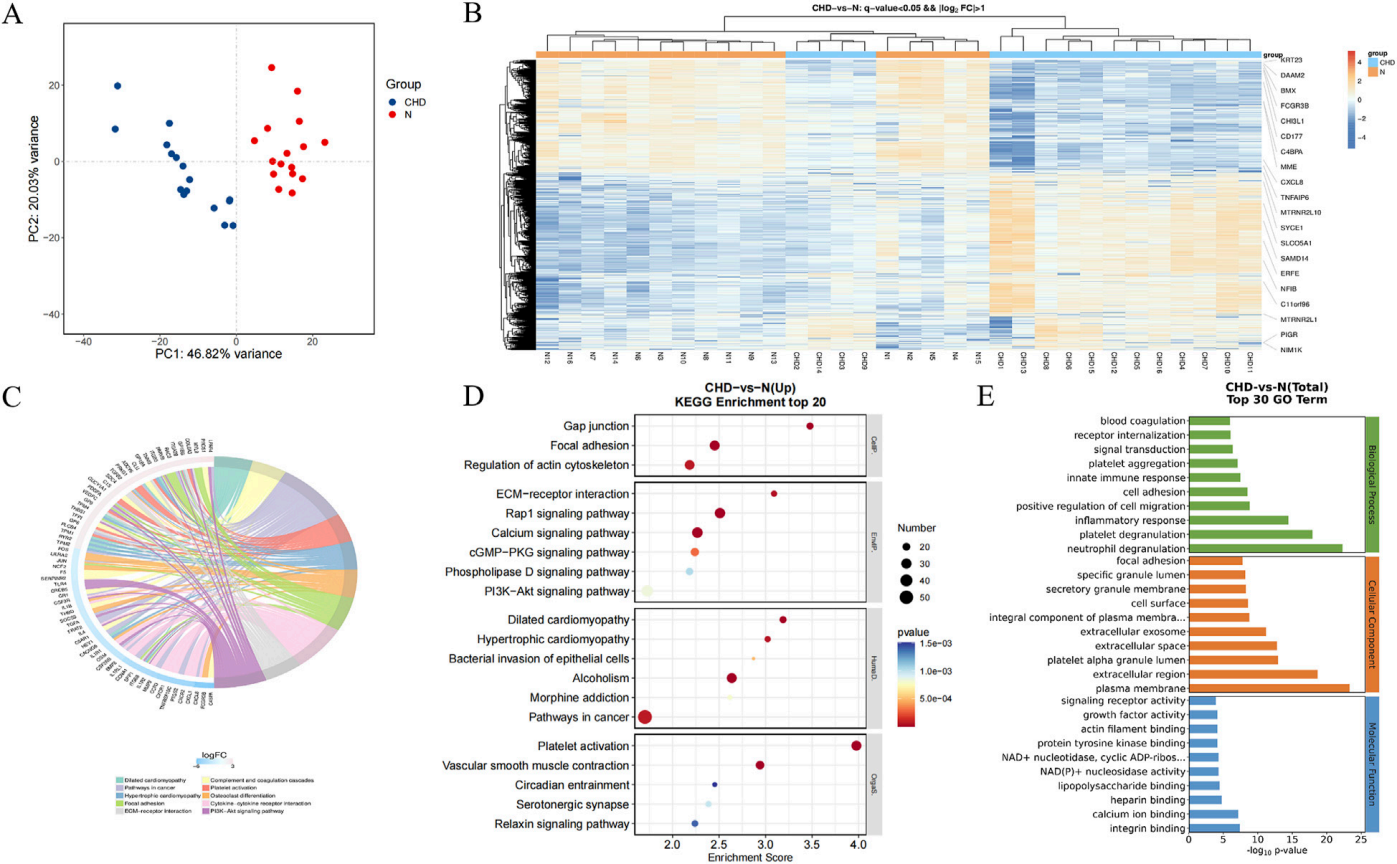
Functional enrichment of platelet genes with differential expression in healthy donors and CHD patients. **(A)**, PCA of control and coronary heart disease samples; **(B)**, heatmaps of differentially expressed genes; **(C)**, hub differentially expressed genes; **(D)**, KEGG enrichment analysis of differentially expressed genes; **(E)**, GO enrichment analysis of differentially expressed genes.

**FIGURE 6:**
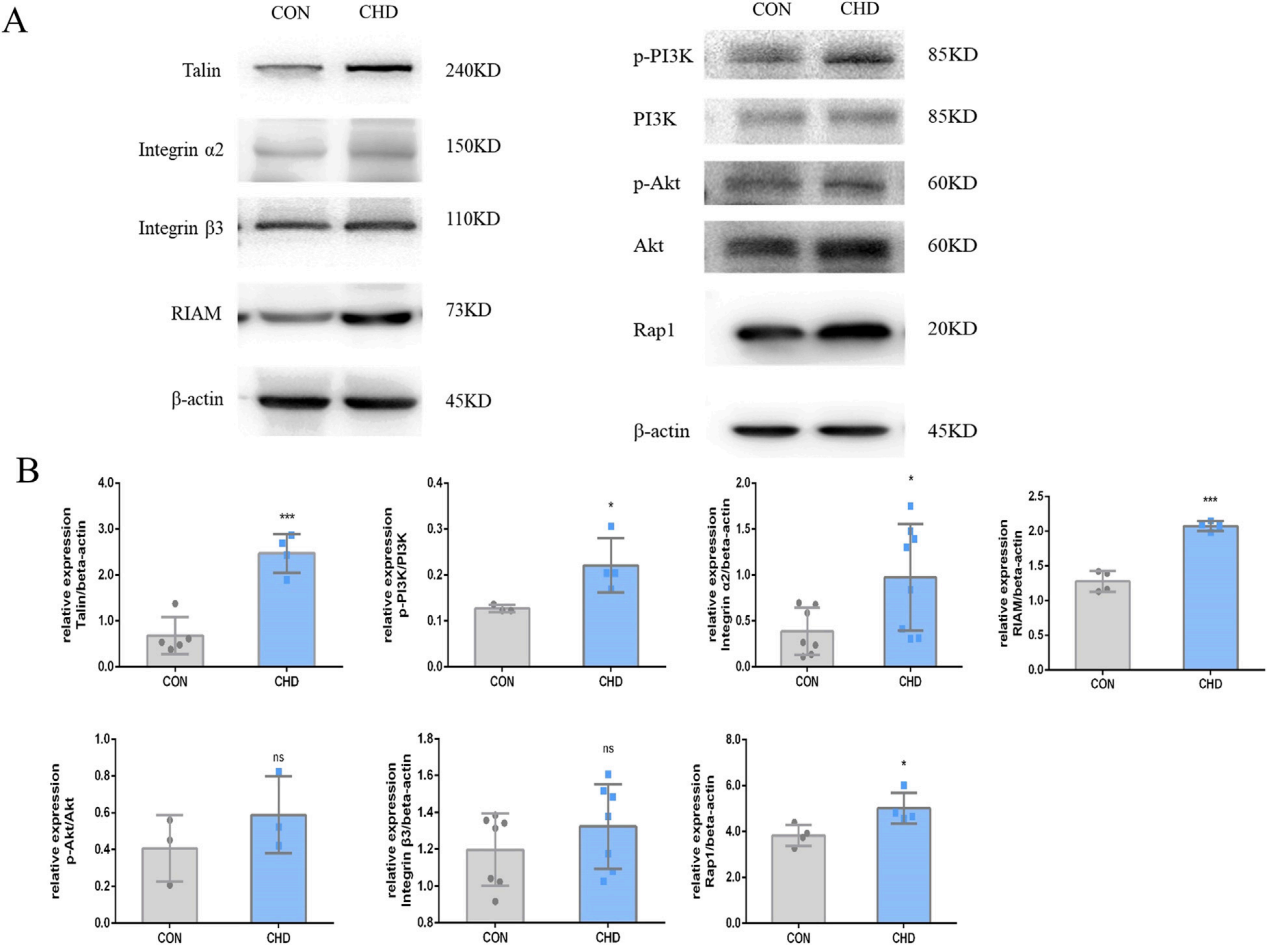
Expression of the Talin-1 and αIIbβ3-mediated bidirectional signaling pathway-related proteins in platelets from CHD patients. **(A)**, Expression of Talin, Integrin α2, β3, RIAM, p-PI3K/PI3K, p-AKT/AKT, and RAP1. **(B)**, Quantitative statistics. The data shown are the means ± SDs of the control; *p < 0.05, **p < 0.01, ***p < 0.001.

According to the transcriptome analysis, platelet RAP1 signaling is critical for CHD (Figure 5). DEGs included a total of 1154 gene upregulated and 742 gene downregulated in CHD. Functional enrichment analysis of these DEGs was conducted using bioinformatics methods, revealing strong correlations with the inflammatory response, platelet particle secretion, platelet activation, cell proliferation, and neutrophil degranulation. Additionally, KEGG pathway analysis revealed enrichment of the Rap1 signaling pathway, calcium signaling pathway, PI3K-Akt signaling pathway, and cytokine signaling pathway. Compared with that in healthy donors, the expression of Rap1, Talin, RIAM, PI3K, and Akt was significantly upregulated in the CHD group (Figure 6). Thus, CHD led to increased protein expression of Rap1, p-AKT, and p-PI3k, which are known to participate in the regulation of adhesion and proliferation.

## Discussion

Platelets are the initial cellular responders to injuries of the vascular endothelium. Upon activation, they secrete autocrine and paracrine mediators, which in turn promote the release of these factors from adjacent cells, thereby facilitating their integration into the developing thrombus. The activation of integrins triggers a signaling cascade that enhances the influx of cytosolic calcium (Du et al., 2011). In the course of this activation process, platelets experience morphological alterations and secrete granule-rich substances, which encompass soluble agonists such as adenosine diphosphate (ADP), adhesion molecules, and coagulation factors. The predominant role of these substances is to enhance the thrombotic response (Barrabés et al., 2005). On the surface of platelets, CD62p and PAC-1 are gold markers of platelet activation. Our results demonstrate that the expression of the activated platelet proteins CD62p and PAC-1 increased, while the proportion of CD62p^+^/PAC-1^+^ platelets decreased (Figure 2). The correspondence between different receptor expressions and the activation of platelet signaling pathways is not fully understood.

Changes in platelet morphology and organelles play crucial roles in platelet function. In the healthy platelets (Figure 3), the outer membrane appeared flat, and the structure of the mitochondrial bilayer membrane and the OCS were clear. The outer membranes of the platelets appeared rough, with protruding pseudopodia, and the platelets were aggregated into clusters. In contrast, the number and quality of mitochondria in the platelets of CHD patients was significantly reduced. Mitochondria is the key regulator of reactive oxygen species and procoagulants in platelets, both of which contribute to pathological thrombosis (Xu et al., 2006). During platelet activation, the integrated involvement of glycolysis and oxidative phosphorylation is facilitated by oxidative stress production-dependent signaling. This signaling, induced by collagen, thrombin, and hyperglycemia, leads to mitochondrial dysfunction, promoting thrombosis under oxidative stress-associated pathological conditions (Gawaz, 2004). Platelets release a wide array of factors that play a role in mediating dynamic functions in inflammation. The majority of these bioactive molecules are released from α-granules, which are unique to platelets and contain an incredibly diverse range of cargoes, including integral membrane proteins, procoagulant molecules, chemokines, mitogenic factors, growth and angiogenic factors, adhesion proteins, and microbicidal proteins (Stellos et al., 2010).

Integrins constitute the principal family of adhesion molecules that mediate cell attachment to the extracellular matrix. They are essential for a variety of physiological processes, including thrombosis, inflammation, the invasion and metastasis of cancer cells, and are also crucial for embryonic development (Sun Z. et al., 2019). Biological information can be transmitted bidirectionally across the plasma membrane. Integrin αIIbβ3 is predominantly expressed in platelets and their progenitor cells, and it is essential for platelet function, hemostatic mechanisms, and the pathogenesis of arterial thrombosis (Huang et al., 2019). In this study, activation of the Talin-1 and αIIbβ3-mediated bidirectional signaling pathway was shown to be one of the mechanisms underlying platelet hyperresponsiveness in patients with CHD (Figures 5, 6). Rap1 protein is a key activating protein in integrin receptors, and the latest mechanism study has found that Rap1 can play an integrin physiological function through the binding of Talin and RIAM proteins. Talins and Kindlins constitute two separate families of FERM domain proteins that engage with the cytoplasmic tails of integrins. These proteins are instrumental in the recruitment of cytoskeletal and signal transduction molecules, which are essential for the process of mechanotransduction. Additionally, they function synergistically to activate integrins, resulting in conformational changes within the integrins and increasing their affinity for extracellular ligands (Chen et al., 2019; Aretz et al., 2023; Lu et al., 2022). Western blotting was used to confirm the expression of the DEGs, revealing significant increases in platelet Rap1 and Talin1 protein expression under disease conditions. Interestingly, a recent epigenetic study on CHD demonstrated that the Rap1 signaling pathway becomes activated after 30 days of CHD (Lagarrigue et al., 2020). This finding suggested that Rap1 is not directly related to platelet responses directly stimulated by stress; rather, the activation of this pathway is driven by platelet adaptation to stress and remodeling. Rap1 serves as a central convergence point in the platelet signaling pathway, regulating the binding of Talin1 to integrin β, thereby modulating integrin activation, platelet aggregation, and hemostasis.

Additionally, Rap1 plays a crucial role in thrombosis, platelet secretion, and phosphatidylserine exposure (Stefanini et al., 2018). Moreover, the GTPase RAP serves as an important regulator of cell adhesion and is an abundant signaling molecule in platelets. Mice lacking the RAP1B subtype exhibit deficiencies in platelet integrin activation, reduced secretion of α granules, and activation of the cytoskeletal regulator RAC1. Combined deficiency of Rap1A and Rap1B adversely affects platelet aggregation, spreading, and clot retraction (Xin et al., 2023). The currently used clinical drug tirofiban targets integrin-αII/βIII. However, commonly used clinical antiplatelet drugs carry a risk of bleeding, drug resistance, and tolerance development. The integrin-αII/βIII receptor is the primary surface receptor following platelet activation, and its bidirectional signaling is crucial for maintaining the balance between platelet activation and the resting state (Guan et al., 2023). Its activation is significantly associated with the prognosis of adverse cardiac disease. The specific physiological mechanism underlying this activation warrants further exploration. The development of therapeutic regimens targeting Rap1 and its binding protein Talin (RIAM), such as negative feedback modulation inhibitors, may lead to better outcomes for coronary heart disease treatment.

In addition to their role in hemostasis, platelets are crucial in modulating the inflammatory response. Flow cytometry was employed to assess the expression levels of platelet polyinflammatory cytokines, revealing two notable phenomena in the blood samples of patients with coronary heart disease: 1) a significant activation of platelet integrins, and 2) alterations in the levels of inflammatory cytokines within the interleukin family (Figures 2, 4, 6). A significant amount of research has demonstrated a relationship between interleukin factor levels and the degree of integrin activation in various pathological circumstances. Specifically, exogenous interleukin-8 has been shown to promote cellular invasion and elevate the levels of integrin β3, phosphorylated phosphoinositide 3-kinase (p-PI3K), and phosphorylated protein kinase B (p-Akt), thereby influencing the invasion and metastasis of hepatocellular carcinoma (Sun F. et al., 2019). Within the tumor microenvironment, interleukin-6 and integrin ανβ6 are critical factors that contribute to the accumulation of mutations, thereby facilitating the progression of cancer. Both exogenous and endogenous interleukin-6 (IL-6) promote the migration and invasion of trophoblast cells that linked to the upregulation of the integrin subunits (Liang et al., 2019; Jovanović and Vićovac, 2009). Interleukin 1 is recognized as a potent bioactive mediator of acute lung injury. Inhibition of the alphavbeta6 integrin and transforming growth factor-beta (TGF-β) mitigates the acute lung injury induced by IL-1beta through RhoA/alphavbeta6 integrin-dependent pathways, with the blockade of the alphavbeta6 integrin effectively preventing IL-1beta-induced protein permeability across alveolar epithelial cell monolayers. *In vivo* investigations revealed that pretreatment with blocking antibodies targeting both the alphavbeta5/6 integrins produced an additive protective effect against IL-1beta-induced acute lung injury (Ganter et al., 2008). The NLRP3 inflammasomes within platelets facilitate the secretion of interleukin-1β during platelet activation and thrombosis *in vitro*. The transfusion of NLRP3-deficient platelets into wild-type mice leads to an extended tail bleeding time and a delay in arterial thrombosis and clot retraction on fixed fibrinogen, which are associated with reduced phosphorylation of c-Src, Syk, and PLCγ2 in response to thrombin stimulation (Qiao et al., 2018). Integrin α5β1 functions as a mechanoreceptor, and its activation through mechanical stimulation initiates a signaling cascade involved the activation of extrusion-activated ion channels, the tyrosine phosphorylation of focal adhesion kinase and paxillin. Subsequently, interleukin-4 is secreted and exerts its effects in an autocrine manner via type II receptors. This process leads to membrane hyperpolarization (Salter et al., 2001). Interleukin-13 (IL-13) and interleukin-17A (IL-17A) engage the type I and IL-17 cytokine receptor families, respectively. Thereby promoting the adhesion of airway smooth muscle cells and facilitate the activation of β1 integrins (Ngo et al., 2024). Among the key molecules investigated for their associative roles in plaque instability are inflammatory biomarkers, such as cytokines and adhesion molecules, lipid-related markers such as oxidized low-density lipoprotein (LDL), and proteolytic enzymes that can degrade extracellular matrix components (Miceli et al., 2024). Previous research conducted by our team has confirmed that platelet-related biological indicators, such as the platelet aggregation rate and the collagen index, can be utilized to evaluate the prognosis and progression of patients with large artery atherosclerotic stroke (Zhan et al., 2024). The rupture of unstable plaques following atherosclerosis is a common cause of myocardial infarction. In another study conducted by our team, platelets from patients with atherosclerosis were collected for transcriptome analysis. It was discovered that the growth factors secreted by platelets are associated with the degree of atherosclerosis, which may have implications for clinical prediction. In all, these studies elucidate a novel function of interleukins in platelet activity, suggesting a new potential connection between thrombosis and inflammation. Consequently, therapies aimed at interleukin may offer therapeutic benefits in the management of inflammation-related thrombosis.

In addition, this study has numerous aspects that require further refinement and should be discussed in subsequent research. Firstly, we have observed mitochondrial changes in the platelets of coronary heart disease patients. However, the exact mechanism by which these changes influence platelet function and thereby regulate thrombus formation remains to be investigated in depth. Existing literature indicates that this may be related to ion transport proteins on the mitochondrial membrane, which regulate mitochondrial energy metabolism and apoptosis (Ghatge et al., 2023; Flora et al., 2023). In animal models, treatments targeting platelet mitochondria have been shown to reduce the risk of thrombus formation (Ajanel et al., 2023; Camacho-Encina et al., 2024). Nevertheless, the functions of platelet mitochondria have not been fully elucidated and there is also a lack of targeted treatment regimens in clinical treatment. Secondly, it remains unclear how changes in platelet genes affect protein expression, which metabolic molecules influence the regulation of platelet functional activity, and what the core regulatory points of numerous accompanying phenomena are. It is still necessary to further explore these aspects using multi-omics techniques. In addition, the number of cases included in this study is relatively small, which may lead to analytical biases. To systematically explore the patterns of platelet abnormalities in coronary heart disease, it is necessary to expand the sample size for further research. Our research team has already conducted preliminary studies on related work, with the aim of further clarifying the disease development patterns.

## Conclusion

Abnormal transcription and platelet activation occur after CHD accompanied by significant morphological changes in platelet organelles, and upregulation of the Talin-1 and αIIbβ3-mediated bidirectional signaling pathway are the main pathological features.

## Data Availability

The data presented in the study are deposited in the NCBI repository, accession number PRJNA1224584.
